# Children with paralytic poliomyelitis: a cross-sectional study of knowledge, attitudes and beliefs of parents in Zamfara state, Nigeria

**DOI:** 10.1186/1471-2458-12-888

**Published:** 2012-10-22

**Authors:** Omoyemi O Ogwumike, Bashir Kaka, Ade F Adeniyi

**Affiliations:** 1 Department of Physiotherapy, College of Medicine, University of Ibadan, Ibadan, Nigeria; 2 Department of Physiotherapy, King Fahd Ibn Abdul-Aziz, Women and Children Hospital, Samaru, Gusau, Zamfara State, Nigeria

**Keywords:** Paralytic poliomyelitis, Knowledge, Beliefs, Attitude, Parents, Nigeria

## Abstract

**Background:**

Nigeria is one of the major African countries in which incidences of polio infection persist in spite of several eradication efforts. The preponderance of paralytic poliomyelitis particularly in the northern part of Nigeria raises the question as to whether parents of children affected with polio know how polio is contracted and spread, whether having a disabled child affects the parents’ attitude towards these children, and what they believe about poliomyelitis in view of their socio-cultural and belief system in the sub-region. Zamfara State, in the north-west of Nigeria is one of the endemic areas where resistance to the global campaign on polio eradication was very high. Therefore this study was conducted to investigate the knowledge, attitudes and beliefs of parents/primary caregivers of children affected with paralytic poliomyelitis in Zamfara State.

**Methods:**

This study is a cross-sectional survey in which the multistage probability sampling technique was used to randomly select two local government areas in Zamfara State where consenting parents/primary caregivers of children with paralytic poliomyelitis were purposively selected. The knowledge, attitudes and beliefs of parents were assessed with the aid of a 4-part 52-item structured researcher administered questionnaire and the data obtained were analyzed.

**Results:**

Two hundred and seventeen parents/primary caregivers participated in the study. One hundred and forty-two, (65.4%) reported good, 51 (23.8%) reported fair, while 24 (11%) of participants reported poor knowledge of paralytic poliomyelitis. More respondents 120 (55.3%) showed a positive attitude towards children with paralytic poliomyelitis. Younger age (P=0.016) and paid employment (P=0.020) were positively associated with good knowledge of paralytic poliomyelitis. Female gender (P=0.020), higher educational level (P=0.015), being employed (P=0.010) and having from middle to high household income (P=0.016) were positively associated with a positive attitude toward children with paralytic poliomyelitis. Most respondents showed a reasonable belief over the cause of their children’s condition rather than the erroneous traditional belief that paralytic poliomyelitis is caused by spirit forces.

**Conclusions:**

It is of great concern that the good knowledge, positive attitude and reasonable belief by parents/primary caregivers about paralytic poliomyelitis observed in this study did not play a prominent role in preventing susceptibility of children in north-west Nigeria to paralytic poliomyelitis. It is imperative that Nigerian policy makers should device more strategic measures toward the prevention of paralytic poliomyelitis in this sub region.

## Background

Poliomyelitis is an acute viral infection which mainly affects children under five years of age [1] with 1 in 200 infections leading to irreversible paralysis usually in the lower limbs. Down from more than 125 countries in 1988, by 2008, persistent pockets of polio transmission in Northern Nigeria, Northern India, and border between Afghanistan and Pakistan were the focus of the polio eradication initiative [2]. As of May 2010, globally there were 237 reported cases of paralytic polio, 52 in endemic countries and 185 in non-endemic countries. Nigeria had 3 cases, one from Delta State (south-south Nigeria), one from Sokoto State and one from Zamfara State (north-west Nigeria) [2]. Immunization is the most reliable method for poliomyelitis prevention [3] but coverage rates in Northern Nigeria are among the lowest in the world [4]. According to the Global Health Program, [5] Nigeria is the primary African country where the polio problem persists resulting in disability of children. A report from the Global Polio Eradication initiative [6] showed that as of November 2011, Nigeria had 52 cases of the wild polio virus.

In endemic areas, parents play a key role in the after-effect of polio infection on their children. This is because their level of knowledge with respect to susceptibility of their children to poliomyelitis and their beliefs about the cause of the disease may influence their willingness to allow polio immunization for their children, thereby enabling possible resistance to poliomyelitis infection [7-9].

Periodic evaluation of the knowledge, attitude and beliefs in a population group serves as an educational diagnosis in a population and provides an important way to measure changing beliefs and behaviours over time. Information from knowledge, attitude and belief studies on polio may form an important element of the programme that ensures a data-driven, evidence-based approach to communication for polio eradication [10].

Socioeconomic, religious and cultural beliefs as well as access to health care services vary among Nigerian sub-regions. Zamfara State is one of the endemic areas where resistance to polio eradication was very high and cases of wild polio virus (WPV) continue to be found [2]. This study was therefore conducted to investigate whether parents/caregivers know how polio virus is contracted and spread, their attitude toward their children with disability and their beliefs about the condition of their children.

## Methods

### Participants

The participants comprised purposively sampled consenting parents of children with paralytic poliomyelitis. Using a multistage probability sampling technique, two out of three senatorial districts in Zamfara State (east and central) were randomly selected out of which two local government areas from each of these senatorial districts were also randomly selected. Five schools and two hospitals were also randomly selected from each local government area. Schools were selected in order to have access to children with obvious lower limb paralysis. The children were then followed up to their homes to meet their parents to obtain responses to the questionnaires. Similarly, in the selected hospitals, parents who brought their children with paralytic poliomyelitis for physiotherapy management were included in the study. Children with paralytic poliomyelitis who were not enrolled in regular schools and were not being managed in the hospitals were located during ‘immunization plus’ days by research assistants who accompanied the immunization administrators on visits to every home in the selected local government areas in order to have responses to the questionnaires.

Ethical approval for the study was obtained from the University of Ibadan/University College Hospital Health Research Ethics Committee. Written informed consent was obtained from all participants.

### Instruments

The instrument used in this study was a 52-item structured questionnaire (Additional file 1) which consisted of 4 parts:

Additional file 1: Section A contained 15 items seeking socio-demographic information from participants such as age, gender, tribe, marital status, employment status, educational level and household income. Employment status was categorized as unemployed, self-employment, government paid and private paid employment. Self-employed persons were petty traders, artisans and subsistence farmers. Persons in private paid employment as opposed to government workers were employed in firms, companies or other organized private establishments not under the federal government of Nigeria. Household income was categorized as: low, middle, moderate and high, based on the federal government of Nigeria’s former minimum wage of 6,500 Naira (41 US dollars) per month. Respondents’ with low income earned less than twice the minimum wage, those in middle earned about 4 times the minimum wage and those with moderate and high income categories earned from five to twelve times the minimum wage.

Additional file 1: Section B contained 15 close-ended item statements inquiring about knowledge of paralytic poliomyelitis, for example causes, effects and preventive measures. Additional file 1: Section C contained 10 close-ended statements inquiring about attitude toward children with paralytic poliomyelitis. Additional file 1: Section D contained 12 close-ended statements on the beliefs of respondents about children with paralytic poliomyelitis in the context of the culture belief system of the respondents. The questionnaire was developed from interviews of parents of children with paralytic poliomyelitis and from previous studies on knowledge, attitudes and beliefs [11-13].

Content validity of the questionnaire was done by five physiotherapists who are experts in the management of polio patients, one epidemiologist and one sociologist. The questionnaire was then pretested for reliability which was found to be as follows: for the knowledge section, a Cronbach’s alpha of 0.74 is obtained, 0.53 for the attitude section and 0.61 for the belief section. The instrument was then translated into Hausa language by a Hausa language expert in the Department of Nigerian Languages, Bayero University Kano, after which it was back-translated into English and then researcher administered for this study. The back translation was done in order to ensure content validity after original English to Hausa translation process and both languages were used in the data collection process, depending on which was easier for the participant to understand. The knowledge section consisted of YES and NO response categories. Each correct response attracted a score of 1 while an incorrect response attracted a zero score. The total score for the section was 15 and this was scale transformed and expressed as a percentage. Overall score was classified as 0-39% - poor, 40-59% - fair and 60-100% - good. For the attitude section, participants responded to each item on a Likert scale scored from 4 (strongly agree) to 1 (strongly disagree) with possible maximum scores ranging from 10 to 40. The scores on items 32, 34, 35, 36 and 39 were reverse scored. Respondents who had scores ranging from 10 to 25 were classified as having a negative attitude while those with scores greater than 25 and up to 40 were classified as having positive attitude. The belief section also had YES or NO `response categories but responses were scored as percentages based on individual item responses. Analysis of data was done using descriptive statistics and Chi-square tests with alpha set at 0.05.

## Results

### Characteristics of respondents

A total of 217 parents (32 male and 185 female) who were invited to participate in this study agreed, and they all gave their informed consent. They were aged 16 to 64 years, with a mean age of 32.29 ± 9.89 (95% CI 30.97-33.61) years. The majority of the respondents 194 (89.4%) were of the Hausa/Fulani tribe. Most of them were married 202 (93.1%). Respondents with primary school education as the highest educational level formed the largest group 96, (44.2%). Almost half of the respondents were in the low income category 105 (48.4%) and the majority were from polygamous family settings 153 (70.5%). These are as shown in Table 1.

**Table 1:** Characteristics of respondents N = 217

**Variables**	**n**	**%**
**Age (years)**		
16-30	118	54.4
31-45	68	31.3
46-60	24	11.1
61-64	7	3.2
**Gender**		
Male	32	14.7
Female	185	85.3
**Marital Status**		
Single	2	0.9
Married	202	93.1
Others	13	6.0
**Tribe**		
Hausa/Fulani	194	89.4
Yoruba	12	5.5
Igbo	8	3.7
Others	3	1.4
**Highest Educational Level**		
Nil	46	21.2
Primary	96	44.2
Secondary	51	23.5
Tertiary	24	11.1
**Employment Status**		
Unemployed	76	35.0
Self Employed	86	39.6
Paid Employment	55	25.3
**Household Income**		
Low	105	48.4
Middle	94	43.3
Moderate/High	18	8.3
**Family Setting**		
Monogamous	64	29.5
Polygamous	153	70.5

KEY: n = frequency, % = percentage, N= Total n0 of participants.

### Parents’ knowledge of paralytic poliomyelitis

The mean score for knowledge of paralytic poliomyelitis in this study was 62.0 ± 17.3 (95% CI: 59.3 – 64.7). One hundred and forty-two respondents [65.4%, 95% CI: 59.0 - 71.8] had good knowledge as against 51 [23.8% 95% CI: 17.8– 29.1] with fair knowledge, and 24 [11.0%, 95% CI: 6.8% - 15.2%] with poor knowledge of paralytic poliomyelitis. The majority of respondents 179 (82.5%) knew that paralytic poliomyelitis mainly affects children under five years of age, while 76.5% reported poor hygiene as a possible cause of predisposition to poliomyelitis. Furthermore, 81.1% of the respondents acknowledged that children who did not obtain or had incomplete immunization were at risk of poliomyelitis infection. Respondents’ knowledge was significantly associated with age group (P=0.016) and employment status (P= 0.010) but not with educational level (P= 0.367) and household income (P= 0.268) [Table 2].

**Table 2:** Association of age group, educational level, employment status and household income with knowledge of paralytic poliomyelitis

**Variables**	**Knowledge of paralytic poliomyelitis**					
	**Good n (%)**	**Fair n (%)**	**Poor n (%)**	**Total n (%)**	**χ**^**2**^	**P**
**Age group (years)**						
16-30	81 (68.8)	30 (24.4)	7 (5.9)	118 (54.4)	15.60	0.016*
31-45	45 (66.2)	15 (22.1)	8 (11.8)	68 (35.0)		
46-60	12 (50.0)	4 (16.7)	8 (33.3)	24 (10.1)		
61-64	4 (57.1)	2 (28.6)	1 (14.3)	7 (0.5)		
Total	142 (65.4)	51 (23.5)	24 (11.1)	217 (100)		
**Highest educational level**						
Nil	32 (69.6)	6 (13.0)	8 (17.4)	46 (21.2)	6.09	0.192
Primary	65 (67.7)	23 (24.0)	8 (8.3)	96 (44.2)		
Secondary	31 (60.8)	14 (27.5)	6 (11.8)	51 (23.5)		
Tertiary	14 (58.3)	8 (33.3)	2 (8.3)	24 (11.1)		
Total	142 (65.4)	51 (23.5)	24 (11.1)	217 (100)		
**Employment status**						
Unemployed	53 (69.7)	19 (25.0)	4 (5.3)	76 (35.0)	18.75	0.010*
Self Employed	47 (54.7)	22 (25.6)	17 (19.8)	86 (39.6)		
Paid Employment	42 (76.4.)	10 (18.2)	3 (5.5)	55 (25.3)		
Total	142 (65.4)	51 (23.5)	24 (11.1)	217 (100)		
**Household income**						
Low	64 (61.0)	26 (24.9)	15 (14.3)	105 (48.4)	4.56	0.336
Middle	63 (67.0)	23 (24.5)	8 (8.5)	94 (43.3)		
Moderate/High	15 (92.9)	2 (11.1)	1 (5.6)	18 (8.3)		
Total	142 (65.4)	51 (23.5)	24 (11.1)	217 (100)		

KEY: *= significant P value n = frequency, % = percentage, N= Total number of participants.

### Attitude of parents toward children with paralytic poliomyelitis

One hundred and twenty respondents [55.3% 95% CI: 26.0 – 32.8] were found to have a positive attitude while 97 [44.7% 95%CI: 18.2 – 25.0] had a negative attitude toward children with paralytic poliomyelitis. Fifty-nine percent strongly agreed that a child with paralytic poliomyelitis should be accepted as a normal child, 82.9% strongly agreed that they should be allowed to attend school like other children, while 67.3% strongly disagreed with the statement that they should be hidden. From Table 3, respondents’ gender (P=0.02), educational level (P=0.015), employment status (P=0.03) and household income (P=0.017) all exhibited significant association with attitude toward children with paralytic poliomyelitis.

**Table 3:** Association of gender, age-group, employment status and household income with attitude toward children with paralytic poliomyelitis

**Variables**	**Positive attitude n (%)**	**Negative attitude n (%)**	**Total n (%)**	**χ**^**2**^	**P**
**Gender**					
Male	12 (37.5)	20 (62.5)	32 (14.7)		
Female	108 (58.4)	77 (41.6)	185 (85.3)	4.81	0.020*
Total	120 (55.3)	97 (44.7)	217 (100)		
**Educational level**					
Nil	21 (45.7)	25 (54.3)	46 (21.2)		
Primary	56 (58.3)	40 (41.7)	96 (44.2)		
Secondary	35 (68.6)	16 (31.4)	51 (23.5)	10.43	0.015*
Tertiary	16 (66.7)	8 (33.3)	24 (11.1)		
Total	120 (55.3)	97 (44.7)	217 (100)		
**Age group (years)**					
16-30	72 (61.0)	46 (39.0)	118 (54.4)		
31-45	32 (47.1)	36 (52.9)	68 (31.1)		
46-60	12 (59.0)	12 (50.0)	24 (11.1)	3.71	0.294
61-64	4 (57.1)	3 (42.9)	7 (3.2)		
Total	120 (55.3)	97 (44.7)	217 (100)		
**Employment status**					
Unemployed	31 (40.8)	45 (59.2)	76 (35.0)		
Self Employed	55 (64.0)	31 (36.0)	86 (39.6)		
Paid Employment	34 (61.8)	21(38.2)	55 (25.3)	10.02	0.010*
Total	120 (55.3)	97 (44.7)	217 (100)		
**Household income**					
Low income	48 (45.7)	57 (54.3)	105 (48.4)		
Middle income	62 (66.0)	32 (34.0)	94 (43.3)		
Moderate/High	10 (55.5)	8 (44.4)	18 (8.3)	8.22	0.016*
Total	120 (55.3)	97 (44.7)	217 (100)		

**Key:** *= significant P value, n= number of respondents %= percentage of respondents inc= income.

### Parents’ beliefs about children with paralytic poliomyelitis

The traditional claim that children with paralytic poliomyelitis have spiritual problem from witches or evil people was rejected by 64.6% of respondents. Most respondents (77%) strongly agreed that seeking medical help is the best treatment option for children with paralytic poliomyelitis (Table 4).

**Table 4:** Respondents’ beliefs about children with paralytic poliomyelitis

**Item-statements**	**True**	**False**
	**n (%)**	**n (%)**
Children with paralytic polio have spiritual problem with witches and or evil people.	77 (35.5)	140 (64.6)
Children from poor homes usually have para-lytic polio	32 (14.7)	185 (85.3)
Children with paralytic polio should be allowed to die to prevent further transmission.	22 (10.1)	195 (89.8)
Children from religious households do not have paralytic polio.	20 (9.2)	197 (90.8)
Most children infected with polio have no symptoms.	39 (18.0)	178 (82.1)
Children may pass polio infection through their feaces.	132 (60.8)	75 (39.1)
Polio immunization for children can cause other problems for example sterility.	48 (22.1)	169 (77.9)
Only one out of every 150 children infected with polio will become paralyzed.	57 (26.3)	160 (73.8)
Children with paralytic polio lose the strength in their limbs and become weak	71 (32.7)	146 (67.2)
Best treatment option for children with paralytic polio is spiritual healing.	34 (15.7)	183 (84.4)
Best treatment option for children with paralytic polio is trado- medical or alternative therapy.	58 (26.7)	159 (73.3)
Best treatment option for children with paralytic polio is to seek medical help from orthodox medical practitioners in a government hospital.	167 (77.0)	50 (23.1)

## Discussion

### Characteristics of respondents

Mothers formed majority of participants in this study. This might be a reflection of their traditional role in the care of children in most parts of the world [14]. The Hausa/Fulani women who constituted majority of the participants were mostly housewives fully involved in home making and care giving to their children. Respondents were mostly in the age range of 16–30 years–a category of young adults in the reproductive age group [15]. A few of the respondents in the older age group were grandparents who were primary caregivers to some of the children in the study. It is a common practice in Nigeria to leave disabled children in the care of grandparents [16] to allow immediate parents give attention to the care of other children in the home or more time for other engagements. Most respondents were married and were Hausa/Fulani–the predominant ethnic group where the study was carried out. The highest educational level was largely primary school for almost half of the respondents. It is noteworthy that Zamfara State is one of the educationally less-developed states in Nigeria, and females are particularly at a disadvantage because their education is usually cut short by early marriage. As regards employment, the self-employed respondents formed the largest group consisting basically of petty traders, artisans and subsistence farmers. These were closely followed by the unemployed. The respondents therefore mostly belonged to the low-income category indicating a prevailing level of poverty in this study group.

### Parents’ knowledge of paralytic poliomyelitis

Most respondents in this study had good knowledge of paralytic poliomyelitis. This was probably due to communication interventions–a large public health initiative organized by the World Health Organization (WHO), the US Centers for Disease Control and Prevention (CDC), the United Nations Children Fund (UNICEF), the United States Agency for International Development (USAID) [17] and Community Participation for Action in the Social Sector (COMPASS) [6,18] Nigeria to educate parents about the importance of preventing paralytic poliomyelitis. Some of this information has been conveyed through slogans and advertisements using the mass media. Also interpersonal means of communication for health education on poliomyelitis and other childhood killer diseases have also been widely emphasized in Nigeria, especially for women who attend antenatal clinics in government hospitals. These may explain the findings of good knowledge in this study. The age of the respondents was found to be inversely related to knowledge of paralytic poliomyelitis; young adults were more knowledgeable than the elderly ones. This was probably because they were more attentive to the mass media–radio and television [19] than the older ones. However, the lack of significant association between level of education and knowledge of paralytic poliomyelitis may be because the majority of the respondents had low level of education, such that their formal academic education did not affect their knowledge of paralytic poliomyelitis. In addition, the fact that household income was not significantly associated with knowledge of paralytic poliomyelitis may be due to the fact that the majority of respondents in this study were from the low income category, hence they may have been more preoccupied with problems surrounding their socio-economic disadvantages than those associated with their children with paralytic poliomyelitis.

### Parents’ attitude toward children with paralytic poliomyelitis

A predominant positive attitude toward children with paralytic poliomyelitis was observed in this study, irrespective of gender, educational level, employment status and household income. This is an indication that these parents were positively disposed toward their children in spite of their disability and were willing to support them. Most therefore strongly disagreed with the statements that families of children with paralytic poliomyelitis should hide them or that the children should not attend regular schools but rather should have special schools. The inclination toward the request for a special school may be justified because it may enable the use of special facilities and skillful handling of these children taking cognizance of their disabilities in order to enhance their quality of life. This is probably why there are few special schools for the disabled in Nigeria [12] but there’s need for more of such schools to be established.

### Beliefs of parents about paralytic poliomyelitis

Most respondents were positive about seeking medical care for their children by accessing orthodox medical service. This is an obvious change from the traditional African belief that witchcraft and ancestral spirits are the causes of paralytic poliomyelitis [20,21] and that the solutions to this problem should also be sought from spirits and ancestral sources [20,22], a belief which usually delayed early diagnosis, intervention and effective rehabilitation. In fact, Renne, [23] reported that many people in northern Nigeria had discarded their traditional belief about poliomyelitis being a disease from a spiritual source, they now appreciate that it is caused by biological factors and so welcome Western medical treatments. Therefore, the belief pattern observed in this study reflected better knowledge of paralytic poliomyelitis. This evidently demonstrates that changes in beliefs in a community or society are possible over time.

### Limitation of study

A notable limitation in this study, however is that since there are other likely causes of lower-limb paralysis apart from polio, the authors cannot categorically state that all children participants in this study had lower-limb paralysis resulting from polio affectation. Suffice it to say that screening for inclusion in this study involved asking for laboratory investigations confirming polio virus presence from those recruited from the hospitals while others from schools and homes were physically diagnosed after thorough clinical assessment.

## Conclusions

Majority of Hausa/Fulani parents of children with paralytic poliomyelitis in north-west Nigeria had good knowledge and a positive attitude concerning the condition of their children. The observed change in their former erroneous belief was encouraging. However, the researchers found it difficult to explain why these developments did not play a prominent role in preventing susceptibility of children to paralytic poliomyelitis. Further studies may investigate what strategic measures could be put in place by Nigerian policy makers so that the knowledge, attitude and beliefs of parents in this sub-region translate to a positive impact on prevention and subsequent eradication of paralytic poliomyelitis.

## Competing interest

The authors’ declare that they have no competing interests.

## Authors’ contributions

OOO contributed to the study concept and design, supervised the research and edited the manuscript. KB helped in data acquisition and prepared the first draft of the paper. AF assisted in revising drafts of the manuscript. All authors read and approved the final manuscript.

## Authors’ information

OOO is a physiotherapist and a lecturer in the Department of Physiotherapy, College of Medicine, University of Ibadan, Ibadan Nigeria and a clinical instructor at the pediatric unit of the Physiotherapy Department, University College Hospital, Ibadan, Nigeria. BK is a graduate student at the Department of Physiotherapy, College of Medicine, University of Ibadan, Ibadan Nigeria, a physiotherapist and a clinician at the Department of Physiotherapy, King Fahd Ibn Abdul-Aziz, Women and Children Hospital, Samaru, Gusau, Zamfara State, Nigeria. AAF is a physiotherapist and a lecturer in the Department of Physiotherapy, College of Medicine, University of Ibadan, Ibadan Nigeria and a specialist adviser at the Orthopedic unit of the physiotherapy department, University College Hospital, Ibadan, Nigeria.

## Pre-publication history

The pre-publication history for this paper can be accessed here:

http://www.biomedcentral.com/1471-2458/12/888/prepub

## Supplementary Material

Additional file 1 Questionnaire on knowledge, attitudes and beliefs toward paralytic poliomyelitis among parents in Zamfara state Nigeria. Click here for file
