# Supplementary material for: Children with paralytic poliomyelitis: a cross-sectional study of knowledge, attitudes and beliefs of parents in Zamfara state, Nigeria
Source: BMC Public Health. 2012 Oct 22;12:888. doi: 10.1186/1471-2458-12-888 (PMC3487889; doi:10.1186/1471-2458-12-888)
Supplement: Additional file 1 — Questionnaire on knowledge, attitudes and beliefs toward paralytic poliomyelitis among parents in Zamfara state Nigeria. [file 1471-2458-12-888-S1.doc]

**QUESTIONNAIRE ON KNOWLEDGE, ATTITUDES AND BELIEFS TOWARD PARALYTIC POLIOMYELITIS AMONG PARENTS IN ZAMFARA STATE NIGERIA.**

SERIAL NO/CODE ­­---­­­­­­­­­­­­­­­­­­­­­­­­­­­­­­­­­­­­­­­----------------

**SECTION A: SOCIO-DEMOGRAPHIC DATA OF PARTICIPANTS**

**Instructions: Indicate the appropriate response**

1. Age of respondent in years: ( )
2. Sex:Male ( ) Female ( )
3. Marital Status: Single () Married ( ) Divorced ( ) Separated( ) Widowed( ).
4. Tribe: Hausa/Fulani ( ) Yoruba ( ) Igbo ( ) Others ( ).
5. Highest Level of Education: None ( ) Quranic ( ) Primary ( ) Junior

secondary ( ) Senior secondary ( ) Tertiary ( ).

1. Mother’s Occupation: Unemployed ( ) Artisan( ) Petty trading ( ) Peasant

farming ( ) Junior Civil Service ( ) Senior Civil Service( ) Medium scale Business ( ) Big Business ( ).

1. Father’s Occupation: Unemployed ( ) Artisan( ), Petty trading ( ) Peasant

Farming ( ) Junior Civil Service ( ) Senior Civil Service ( ) Medium scale Business ( ) Big Business ( ).

1. Household income per month: Less than ~~N~~12,000( ) ~~N~~ 13,000 – 49,000 ( )

~~N~~50,000 –90,000 ( ) ~~N~~ 100,000 and above ( ).

9. Family setting: Monogamous( ) Polygamous( ).

10. Religion: Christianity ( ) Islam ( ) Others( ).

BIODATA OF THE CHILD

1. Sex:Male ( ) Female( ).
2. Age: (in years) ( )
3. Time since diagnosis of child’s problem: ( ).
4. Have you ever gone to the hospital for medical treatment? Yes ( ) No ( ).
5. Have you ever gone for physiotherapy treatment? Yes ( ) No ( ).

**SECTION B: KNOWLEDGE OF PARENTS ABOUT POLIOMYELITIS**

**Instruction:** The following statements refer to knowledge of poliomyelitis. Mark the boxthat best describes your answer.

16. I know what causesparalytic poliomyelitis Yes No

17. Paralytic Poliomyelitis is caused by accidents Yes No

18. Paralytic Poliomyelitis is caused by a virus and can spread easily among people

Yes No

19. Paralytic Poliomyelitis is sometimes called infantile paralysis because it affects mostly children under 5 years of age. Yes No

20. Symptoms of polio include fever, body weakness, headache and vomiting. Yes No

21. Children who have not been immunized or did not complete immunization are at risk of getting Paralytic Poliomyelitis. Yes No

22. Polio immunization can result in sterility and or HIV infection.

Yes No

23. Infection from polio can cause death of the child. Yes No

24. Polio virus can contaminate food or water. Yes No

25. The polio immunization may either be given as a series of shots or as drops taken by mouth.Yes No

26. Polio is most common where there are no bathroom or latrines or where there

ispoor hygiene .Yes No

27. It is possible to transmit polio from mother to child. Yes No

28. Paralytic Poliomyelitis is curable. Yes No

29. No medicine can cure Paralytic Poliomyelitis.Yes No

30. Weakness of the legs are the most visible signs of poliomyelitis. Yes No

**SECTION C ATTITUDES TOWARD CHILDREN WITH PARALYTIC POLIOMYELITIS**

**Instructions**: The following statements refer to attitude of parents towards children with poliomyelitis infection. Tick the appropriate answers

SA= Strongly agree, A = Agree, D = Disagree, SD = Strongly disagree

| **S/No** | **ITEM** | | **SA** | | **A** | | **D** | **SD** |
| --- | --- | --- | --- | --- | --- | --- | --- | --- |
| 31 | They always need help. | |  | |  | |  |  |
| 32 | They may not be able to lead a normal life. | |  | |  | |  |  |
| 33 | A child with paralytic polio needs to be accepted as a normal child and should freely socialize in the community. | |  | |  | |  |  |
| 34 | A child with paralytic polio is specially ordained by God and nothing can be done about it. | |  | |  | |  |  |
| 35 | He /She needs to be hidden from public because of shame. | |  | |  | |  |  |
| 36 | If severely disabled, they should be taught at home. | |  | |  | |  |  |
| 37 | Regular school will not give adequate attention to them. | |  | |  | |  |  |
| 38 | Children in regular schools will fight and make fun of them. |  | |  | |  | |  |
| 39 | They should not attend school, they should go begging because they are not like other children. |  | |  | |  | |  |
| 40 | They should be allowed to attend schools like any other child to have a brighter future. |  | |  | |  | |  |

**SECTION D BELIEFS OF PARENTS ABOUT PARALYTIC POLIOMYELITIS**

**Instructions: The following statements refer to beliefs of parents about children with paralytic poliomyelitis (PP). Please tick the appropriate response.**

| **S/No** | **ITEM** | **Yes** | **No** |  |
| --- | --- | --- | --- | --- |
| 41 | Children with PP have spiritual problem from witches and or evil people |  |  |
| 42 | Children from poor homes usually have PP |  |  |
| 43 | Children with PP should be allowed to die to prevent further transmission |  |  |
| 44 | Children from religious households do not have PP. |  |  |
| 45 | Most children infected with PP have no symptoms |  |  |
| 46 | Children may pass PP infection through their faeces. |  |  |
| 47 | Polio immunization for children can cause other problems for example sterility. |  |  |
| 48 | Only one out of every 150 children infected with polio will become paralyzed. |  |  |
| 49 | Children with PP lose the strength in their limbs and they become weak. |  |  |
| 50 | Best treatment option for children with PP is spiritual healing |  |  |
| 51 | Best treatment option for children with PP is trado- medical or alternative therapy. |  |  |
| 52 | Best treatment option for children with PP is to seek medical help from orthodox medical practitioners in a government hospital. |  |  |
